# Editorial: 3D printing in pharmaceuticals and medical applications

**DOI:** 10.3389/fddev.2024.1527225

**Published:** 2024-11-26

**Authors:** Italo Rodrigo Calori, Dimitrios A. Lamprou

**Affiliations:** ^1^ Department of Pharmaceutics and Drug Delivery, School of Pharmacy, University of Mississippi, Oxford, MS, United States; ^2^ School of Pharmacy, Queen’s University Belfast, Belfast, United Kingdom

**Keywords:** 3D printing, biomedical applications, drug delivery, tissue engineering, personalized medicine

In the last years, there is an increased interest in 3D printing (3DP) for applications in drug delivery and tissue engineering, with the potential of the systems to be used also in space explorations for in demand and inhouse manufacturing. 3DP is an innovative and rapidly evolving technology that is revolutionizing the biomedical field, and not only. By enabling the creation of personalized biomedical devices, implants, tissue scaffolds, and drug delivery systems, it meets the growing demand for individualized treatments. 3DP is a layer-by-layer (LbL) deposition process, which allows for precise control over material properties and structures from the macroscopic to the microscopic level. This level of precision overcomes the limitations of traditional manufacturing methods, opening new possibilities for patient-specific solutions that are both effective and cost-efficient. The ability to create advanced, customized devices is transforming medicine, particularly in drug delivery and regenerative therapies.

This Research Topic presents the latest developments in 3DP for biomedical applications, encouraging further innovation and exploration in the field.

Levine et al., investigated how the geometry of laser-sintered tablets containing theophylline affects their properties. Their study focused on key characteristics such as microstructure, volume, disintegration, and dissolution. The authors, tested tablets in three distinct shapes, cylindrical, hollow cylindrical, and frustum, while keeping the surface area constant and varying the cylinder diameters. Advanced characterisation methods, such as Micro-computed tomography (Micro-CT) analysis revealed how these geometric changes influenced tablet properties such as mass uniformity and disintegration, providing valuable insights for drug formulation.

Guimaraes et al., introduced the SpheroMold, a 3D-printed device designed to improve the hanging drop method for spheroid culture. By utilizing stereolithography, they printed a negative mould from photopolymer resin and filled it with Polydimethylsiloxane (PDMS) to create a support for 3D cell culture using the hanging drop method. This device reduced drop coalescence and expanded the culture medium volume, enhancing spheroid formation and improving process efficiency.

In another study, De Vincentiis et al., developed a 3D-printed wearable device for mouse models with spinal cord injury. The authors aimed preparing a non-invasive method and prioritizing animal welfare. The paper provides tests of various formats, including “Fitbit,” “Belt,” “Bib,” and “Cape,” on both healthy and injured mice. The effectiveness of these devices was demonstrated through simulations using the finite element method and magnetic field measurements, showing promising results for spinal injury treatment.

Lastly, Schweiker et al., explored the potential of 3D-printed biodegradable scaffolds for bone regeneration. They combined an alginate core with an outer layer of calcium phosphate cement and employed a modified bioprinter to print the scaffolds. Post-printing treatments such as drying, PBS incubation, and lyophilization were applied to assess the scaffolds’ mechanical strength. Mechanical testing and scanning electron microscopy analysis confirmed the scaffolds’ robustness and potential in tissue engineering and bone regeneration.

In conclusion, these studies highlight the transformative potential of 3DP in various biomedical fields, from drug formulation to tissue engineering. Levine et al.'s investigation of laser-sintered tablets underscores the importance of geometry in drug design. The SpheroMold technology illustrates how 3DP can enhance traditional spheroid culture methods. The wearable device for spinal injury models addresses key concerns such as non-invasive approaches and animal welfare. Finally, Schweiker et al., work on biodegradable scaffolds advances tissue engineering and bone regeneration. These innovations not only enhance scientific understanding but also pave the way for safer, more effective therapies.

The editors, sincerely thank the authors for their valuable contributions, the reviewers for their constructive feedback, and the editorial staff of *Frontiers in Drug Delivery* for their professional support. We hope this Research Topic will inspire continued research and development in the field of 3DP in the era of biomedical applications and beyond.

